# Evaluation of the antiproliferative effects of the HASPIN inhibitor CHR-6494 in breast cancer cell lines

**DOI:** 10.1371/journal.pone.0249912

**Published:** 2021-04-14

**Authors:** Hisayo Nishida-Fukuda, Keizo Tokuhiro, Yukio Ando, Hiroaki Matsushita, Morimasa Wada, Hiromitsu Tanaka

**Affiliations:** 1 Department of Genome Editing, Institute of Biomedical Science, Kansai Medical University, Hirakata City, Osaka, Japan; 2 Faculty of Pharmaceutical Sciences, Nagasaki International University, Sasebo, Nagasaki, Japan; Virginia Tech, UNITED STATES

## Abstract

HASPIN is a serine/threonine kinase that regulates mitosis by phosphorylating histone H3 at threonine 3. The expression levels of HASPIN in various cancers are associated with tumor malignancy and poor survival, suggesting that HASPIN inhibition may suppress cancer growth. As *HASPIN* mRNA levels are elevated in human breast cancer tissues compared with adjacent normal tissues, we examined the growth-suppressive effects of CHR-6494, a potent HASPIN inhibitor, in breast cancer cell lines *in vitro* and *in vivo*. We found that *HASPIN* was expressed in breast cancer cells of all molecular subtypes, as well as in immortalized mammary epithelial cells. *HASPIN* expression levels appeared to be correlated with the cell growth rate but not the molecular subtype of breast cancer. CHR-6494 exhibited potent antiproliferative effects against breast cancer cell lines and immortalized mammary epithelial cells *in vitro*, but failed to inhibit the growth of MDA-MB-231 xenografted tumors under conditions that have significant effects in a colorectal cancer model. These results imply that CHR-6494 does have antiproliferative effects in some situations, and further drug screening efforts are anticipated to identify more potent and selective HASPIN inhibition for use as an anticancer agent in breast cancer patients.

## Introduction

Haploid germ cell-specific nuclear protein kinase (HASPIN), also known as germ cell-specific gene 2 (GSG2), was first discovered in germ cells of male mice and is highly conserved in eukaryotic organisms. HASPIN possesses serine/threonine kinase activity and has been shown to phosphorylate histone H3 [1–4]. Phosphorylation of histone H3 at threonine 3 (pH3T3) by HASPIN is necessary for chromosomal passenger complex accumulation at centromeres, which is required for correct spindle-kinetochore attachment during chromosome segregation and cytokinesis [5–7]. RNA interference-mediated knockdown of HASPIN has been shown to inhibit cell proliferation and cause cell cycle arrest at the G2/M phase, thereby inducing apoptosis of pancreatic cancer cells [8]. Given that HASPIN expression is restricted to proliferating cells [9], HASPIN inhibition could be a viable strategy for cancer treatment. Of note, HASPIN was found to be upregulated in a wide range of cancers, and high HASPIN expression levels in tumors have been associated with a poor patient prognosis [8, 10–12]. More recently, HASPIN upregulation has been reported during gallbladder carcinoma (GBC) progression, and HASPIN silencing by shRNA inhibited proliferation of GBC cells [11].

Several HASPIN inhibitors have been developed; these agents include acridine derivatives, beta-carboline derivatives, ribofuranosyl derivatives, ARC-type bisubstrate inhibitors, CX-6258, and CHR-6494 [13–17]. 5-Iodotubercidin (5-ITu), which was initially classified as an adenosine kinase inhibitor, is another commonly used for HASPIN inhibitor [18, 19]. Although low concentrations of 5-ITu inhibit H3T3 phosphorylation [17, 20, 21], *in vitro* analysis of *HASPIN*-deficient cells generated by CRISPR/Cas9 indicated that 5-ITu has off-target effects [18]. CX-6258, an orally administered pan-Pim kinase inhibitor, has recently been shown to inhibit HASPIN and reduce proliferation of melanoma cell lines (A375-S and A375-RMR) both *in vitro* and *in vivo* [10]. Similarly, CHR-6494, a potent HASPIN inhibitor, has been demonstrated to inhibit proliferation of the triple-negative breast cancer cell line MDA-MB-231 [22] and various melanoma cell lines *in vitro* [23].

Breast cancer is the most frequent malignancy in women and an extremely heterogeneous disease at the molecular level. Based on the receptors they express, breast tumors are classified into four major molecular subtypes: luminal A, luminal B, HER2-enriched, and triple-negative/basal-like [24]. Compared with estrogen receptor (ER)-positive subtypes, triple-negative/basal-like tumors are often more aggressive and have a poor prognosis [25]. A recent study revealed that in all four subtypes of breast cancer tissue, mRNA levels of *HASPIN* were significantly higher than in corresponding adjacent normal tissues, suggesting that HASPIN is a potential target for breast cancer therapy [12]. Although HASPIN appears to play an important role in cancer progression, its role in breast cancer development and progression remains largely unknown. In this study, we examined the *in vitro* and *in vivo* effects of the HASPIN inhibitor CHR-6494 on different breast cancer cell lines.

## Results

### HASPIN is broadly expressed in cancer and normal breast cell lines

**Fig 1:**
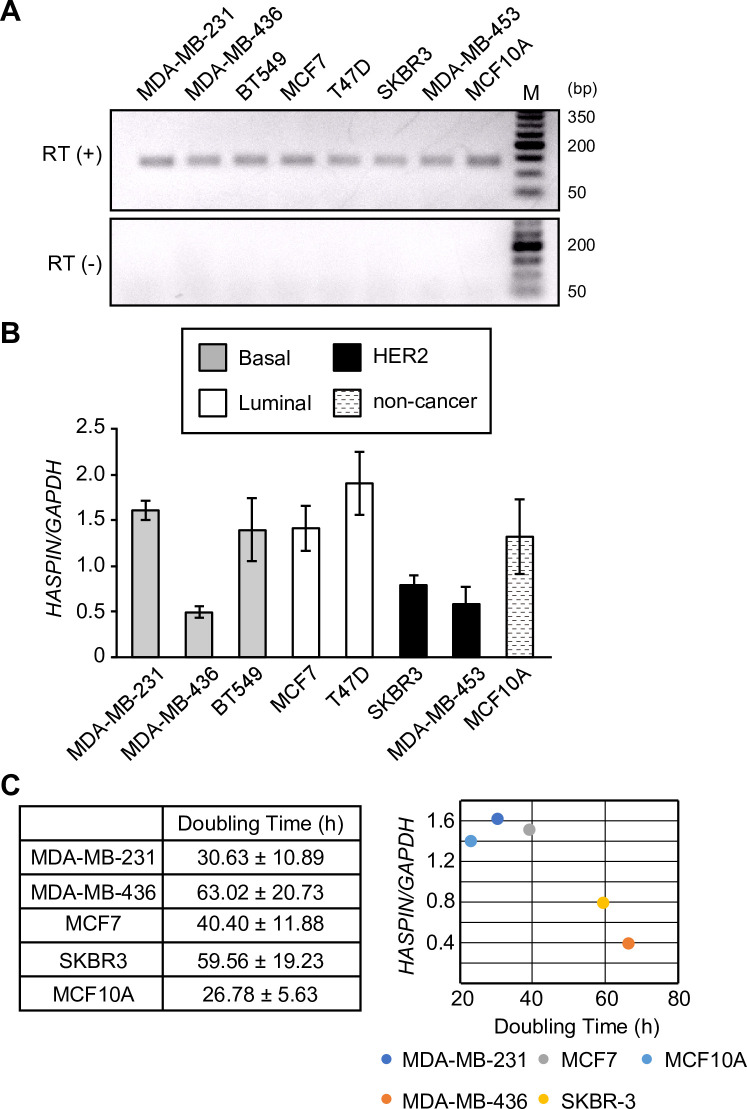
*HASPIN* mRNA levels in breast cancer cell lines. (A) Agarose electrophoresis confirming the specificity of the primer pair in RT (+) (top) and the absence of genomic DNA contamination in RT (−) (bottom). (B) Quantification of *HASPIN* mRNA levels in breast cancer cell lines and MCF10A cells. Data are presented as the mean ± SD of three independent experiments. (C) Doubling times ± SD (hours) of breast cancer cells and MCF10A cells (left panel). Data are presented as the mean ± SD of three independent experiments. Pearson’s correlation coefficient between *HASPIN* expression level and doubling time in breast cancer cells (r = −0.913, *P* < 0.05, right panel).

**Fig 2:**
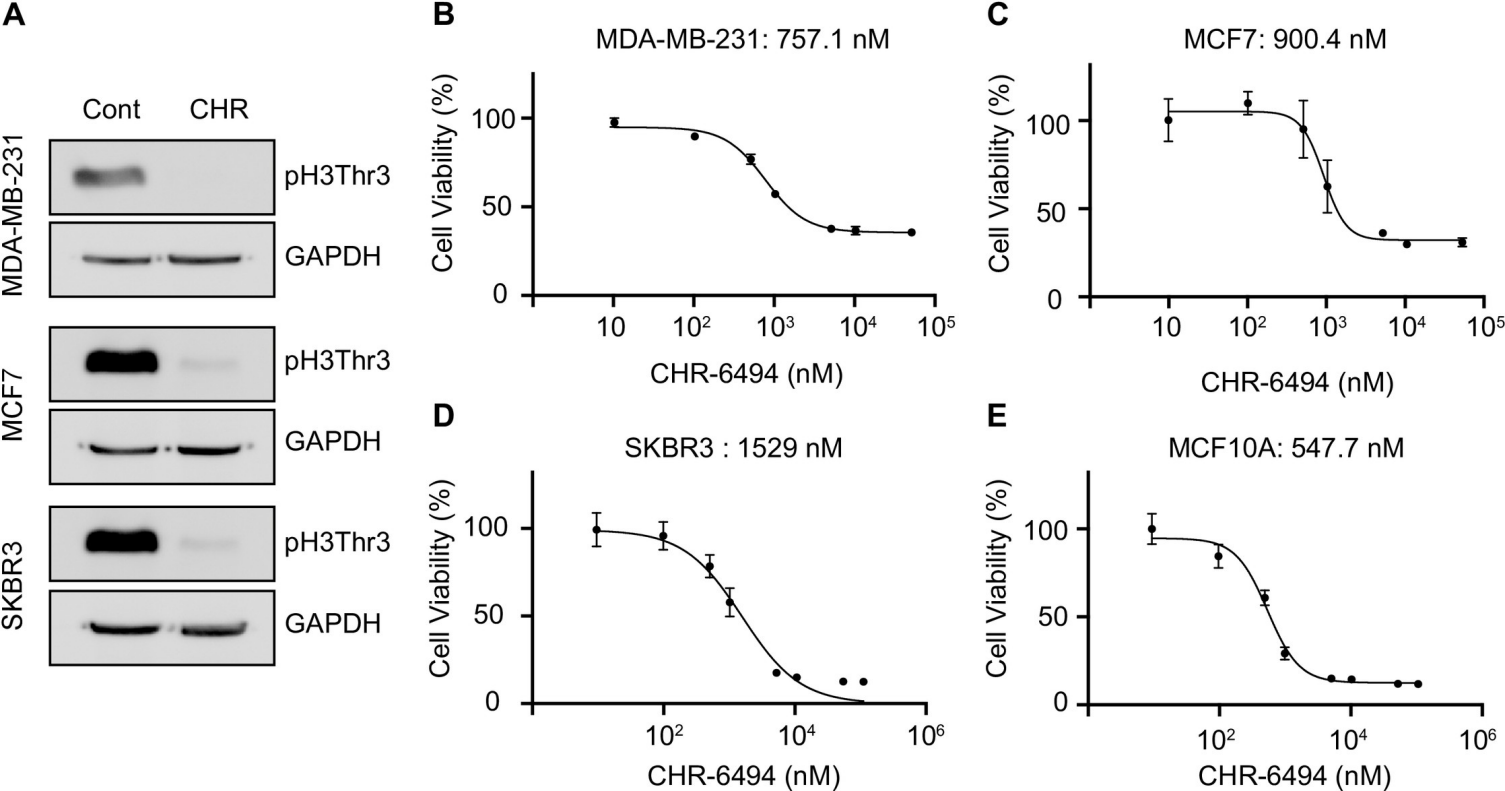
CHR-6494 inhibits the proliferation of breast cancer cells *in vitro*. (A) Immunoblotting analysis of pH3T3. The breast cancer cells were treated with DMSO or CHR-6494 (1000 nM) for 24 h. The effects of CHR-6494 on the viability of MDA-MB-231 (B), MCF7 (C), SKBR3 (D), and MCF10A (E) cells were determined by XTT assay. IC_50_ values are shown for each cell line. Data are presented as the mean ± SD of three independent experiments.

Previous studies have shown that HASPIN is broadly and highly expressed in various cancers compared with the corresponding non-malignant tissues [10, 11, 26]. A recent study has also demonstrated that among the four breast cancer subtypes, luminal A breast cancer tissues had the lowest *HASPIN* expression levels, whereas basal-like breast cancer tissues exhibited the highest *HASPIN* expression [12]. To confirm the mRNA levels of *HASPIN* in different breast cancer types, we performed quantitative real-time PCR (qRT-PCR) analysis using several breast cancer cell lines and MCF10A cells, which are immortalized human mammary epithelial cells commonly used as a normal breast cell model. Since *HASPIN* is an intronless gene, the PCR primer pair could not distinguish between cDNA and genomic DNA. Reverse transcriptase (RT)-negative samples exhibited no amplification products (Fig 1A, bottom panel), confirming the absence of genomic DNA contamination. *HASPIN* was expressed in normal mammary epithelial cells and all of the breast cancer cell lines tested, and the expression levels of *HASPIN* were not associated with any breast cancer subtypes (Fig 2B). Although HASPIN is expressed in proliferating cells but not in non-dividing cells [9], the current knowledge of HASPIN function does not provide a mechanism by which the elevated expression of HASPIN would lead to enhance cell proliferation. Therefore, there is a possibility that HASPIN expression in breast cancer tissues is associated with the number of proliferating cells. To examine whether there is a correlation between the *HASPIN* expression level and growth rate of breast cancer cells, we next examined the growth rates of the breast cancer cell lines (Fig 1C, left). The doubling time of cells was negatively correlated with the level of *HASPIN* expression (r = -0.913, P<0.05, Fig 1C, right). These results imply that, at least *in vitro*, expression levels of HASPIN may be associated with the rate of cell proliferation, but not with the breast cancer subtype.

### CHR-6494 inhibition of HASPIN suppressed breast cancer cell growth *in vitro*

As HASPIN regulates cell cycle progression [13, 27], HASPIN inhibition may block mitosis in breast cancer cells. Previous reports have shown that HASPIN inhibitors have antiproliferative activity and induce apoptosis of melanoma, colon carcinoma, and pancreatic cancer cells [8, 22, 23, 28]. CHR-6494 is a commercially available agent and has antiproliferative effects on xenografted tumor formation [22, 28]. In this study, we assessed the effects of CHR-6494 on different breast cancer cell lines and MCF10A cells. Consistent with previous reports, CHR-6494 treatment substantially diminished pH3T3 levels in breast cancer cells (Fig 2A). Next, cell viability after CHR-6494 treatment was assessed using the XTT (sodium 3´-[1-(phenylaminocarbonyl)-3,4-tetrazolium]-bis (4-methoxy6-nitro) benzene sulfonic acid hydrate) assay, as described previously [22]. CHR-6494 inhibited cell growth in a dose-dependent manner, with half-maximal inhibitory concentration (IC_50_) values of 757.1 nM for MDA-MB-231, 900.4 nM for MCF7, and 1.53 for SKBR3 (Fig 2). The IC_50_ of CHR-6494 in MCF10A cells was 547 nM, which was the lowest among all the cell lines tested. This finding suggested that although CHR-6494 exerts an antiproliferative effect on breast cancer cells, this effect is not related to the malignancy of breast cancer.

**Fig 3:**
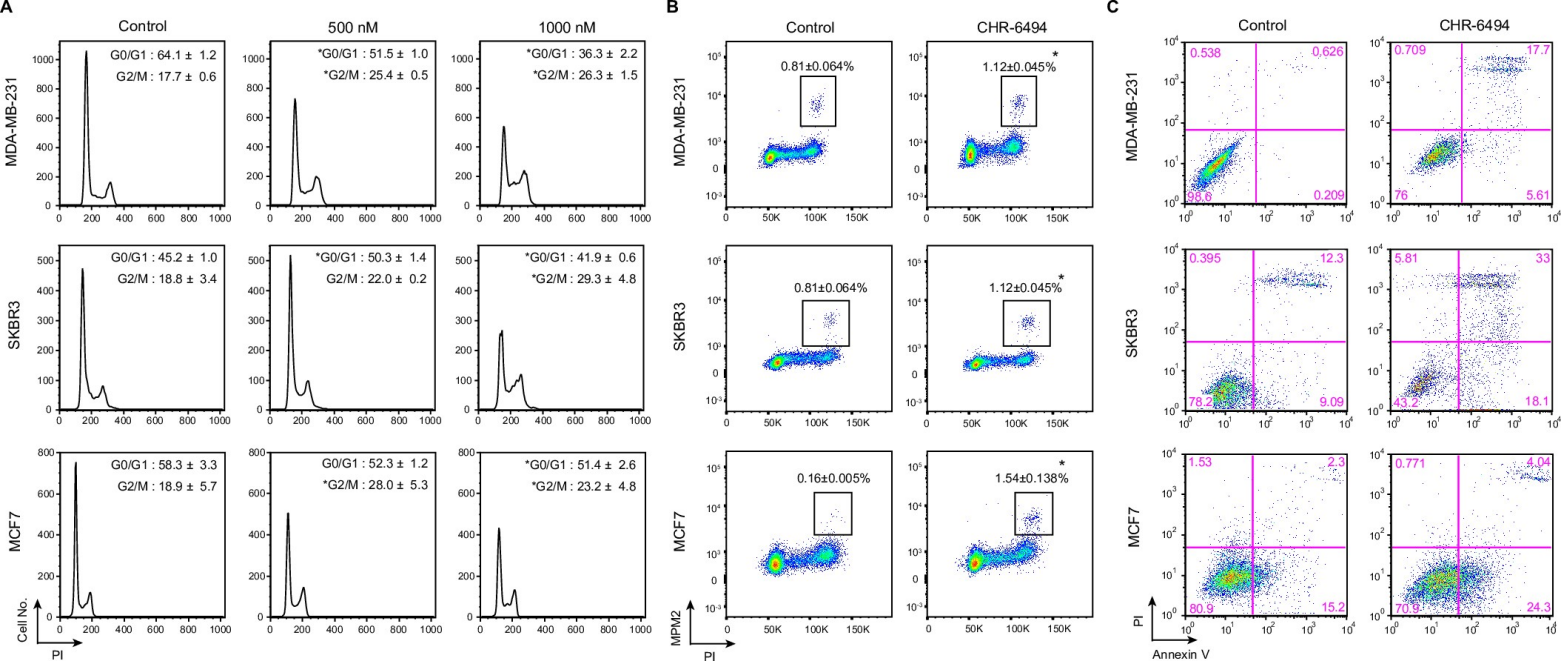
CHR-6494 induces cell cycle arrest and apoptosis *in vitro*. (A, B) Flow cytometry analysis of control and CHR-6494-treated breast cancer cells. DNA profiles (A) and phospho-MPM-2 profiles (B). Black boxes indicate the gated mitotic cell populations (B). (C) Dot plots showing the distributions of FITC-Annexin V/PI-stained control and CHR-6494-treated breast cancer cells. The cells in the lower left quadrant represent viable cells (FITC-Annexin V/PI-negative). The cells in the upper and lower right quadrants represent the late and early apoptotic cells, respectively. Data are presented as means ± SD of three independent experiments. *P < 0.05 (Student’s *t* test).

Many studies have shown that HASPIN inhibitors have the ability to induce cell cycle arrest and apoptosis [22, 23, 27]. To examine the effects of CHR-6494 on the cell cycle in breast cancer cells, we assessed the distribution of cells in G0/G1, S, and G2/M phases by flow cytometry after staining cells with propidium iodide (PI). In MDA-MB-231 cells, 500 nM and 1000 nM CHR-6494 increased the percentage of cells in G2/M phase from 17.7 ± 0.6% to 25.4 ± 0.5% and 26.3 ± 1.5%, respectively (Fig 3A). Similar results were observed in SKBR3 and MCF7 cells with the exception of SKBR3 cells treated with 500 nM CHR-6494 (*P* = 0.059). The increase in the percentage of cells in G2/M phase was accompanied by a decrease in the percentage of cells in G0/G1 phase. To determine the percentage of cells in M phase, cells were stained with PI and anti-phospho-Ser/Thr-Pro MPM-2 antibody. As shown in Fig 3B, CHR-6494 treatment at 1000nM significantly increased MPM-2 positive cells compared with control. However, this increase in MPM-2 positive (mitotic) cells cannot fully explain the increase in the fraction of G2/M cells resulting from CHR-6494 treatment (Fig 3A), indicating that the majority of arrested cells would be in G2. These results are consistent with previous reports indicating that HASPIN depletion leads to cell cycle arrest at both interphase and mitosis [8, 19, 29]. To examine the effects of CHR-6494 on apoptosis, we stained cells with Annexin V-fluorescein isothiocyanate (FITC)/PI. In MDA-MB-231 and SKBR3 cells, CHR-6494 significantly increased the percentage of cells undergoing late and early apoptosis; however, the effects of CHR-6494 on MCF7 cell apoptosis were less profound (Fig 3C). These results show that CHR-6494 induces G2/M arrest and apoptosis in breast cancer cells in a dose-dependent manner.

### Effects of CHR-6494 on breast cancer growth *in vivo*

**Fig 4:**
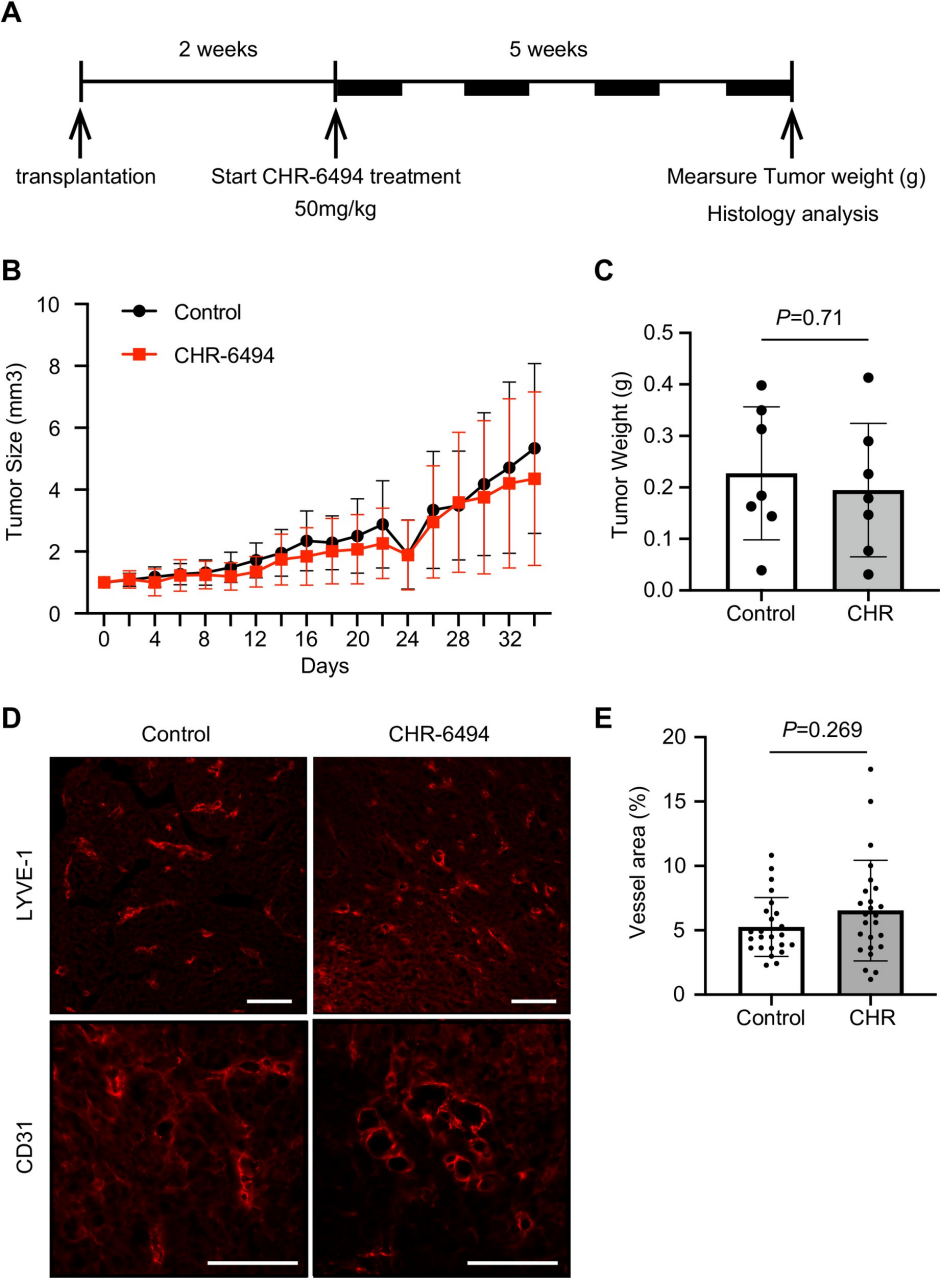
Effects of CHR-6494 on the growth of MDA-MB-231 xenografts. (A) Schematic representation of CHR-6494 treatment in MDA-MB-231 xenografted mice. Two weeks after MDA-MB-231 transplantation in immunodeficient nude mice, mice received four cycles of CHR-6494 (50 mg/kg) injected intraperitoneally (5 consecutive days over 35 days). (B) Tumor volume (mm^3^) was monitored in control and CHR-6494-treated mice. Data are presented as the means ± SD (n = 7 mice). (C) Tumor weight (g) was measured at the study endpoint. *P* = 0.71; Mann–Whitney U test. Data are presented as the mean ± SD (n = 7 mice). (D) Immunofluorescence of CD31-positive (top) and LYVE-1-positive (bottom) vessels in MDA-MB-231 tumors. Scale bars: 100m. (E) Vessel area of the tumors (LYVE-1-positive). *P* = 0.269; Student’s *t* test. Data are presented as the mean ± SD (n = 4 mice).

Given that CHR-6494 has been reported to inhibit the growth of human colorectal cancer cells *in vivo* [22, 28], we examined the antitumor activity of CHR-6494 in breast cancer using an MDA-MB-231 xenograft mouse model. Two weeks after orthotopic injection of MDA-MB-231 cells (average tumor volume of 62 mm^3^), mice were randomly divided into control (vehicle-treated; n = 7) and CHR-6494 (50 mg/kg) treatment (n = 7) groups. Mice received four cycles (5 consecutive days over 35 days) of intraperitoneal injection with CHR-6494 or vehicle (Fig 4A). In contrast to previous findings, CHR-6494 did not affect the growth of MDA-MB-231 tumors (Fig 4B and 4C). CHR-6494 has been reported to inhibit tumor-associated angiogenesis [22]; hence, we examined tumor-associated vasculogenesis by staining MDA-MB-231 tumors for CD31 and LYVE-1 (Fig 4D). We found no significant difference in the extent of tumor-associated angiogenesis (indicated by CD31) or lymphangiogenesis (indicated by LYVE-1) between CHR-6494-treated and vehicle-treated mice (Fig 4D and 4E). These results imply that CHR-6494 (at 50 mg/kg) cannot inhibit tumor growth in the MDA-MB-231 xenograft mouse model.

## Discussion

HASPIN is required for histone H3 phosphorylation at threonine 3 during mitosis, and treatment with CHR-6494 substantially decreases pH3T3 levels *in vitro* [8, 19, 22]. Our *in vitro* data suggest that CHR-6494 has antiproliferative effects on breast cancer cell lines and normal mammary epithelial cells. However, CHR-6494 treatment failed to suppress the growth of MDA-MB-231 tumors in our breast cancer xenograft mouse model. One possible explanation for the discrepancy between *in vitro* and *in vivo* findings is that other kinases compensate for the loss of HASPIN function *in vivo*, which is supported by the fact that *Haspin*-deficient mice are fertile and have no obvious phenotype, despite the importance of HASPIN for mitosis [30]. One compensatory candidate is vaccinia-related kinase 1 (VRK1), which phosphorylates histone H3 at threonine 3 and serine 10 [31]. VRK1 is widely expressed in proliferative tissues, including embryonic tissues, adult testis, and thymus, as well as in several cancer cell lines [32]. In addition to histone H3, VRK1 phosphorylates several transcription factors, including p53, c-Jun, and ATF2. Moreover, VRK1 positively regulates the expression of *BIRC5* (Survivin), which recognizes pH3T3 [33, 34]. Unlike *Haspin-*deficient mice, VRK1 hypomorphic mice are infertile [35]. A recent report showed that VRK1 interacts with Aurora B, inhibiting the kinase activity of the latter [34]. Aurora B phosphorylates HASPIN, thereby increasing the levels of pH3T3 [36]. These findings support that VRK1 may compensate for the loss of HASPIN function; future studies of the spatiotemporal regulation of these kinases are required to elucidate their interplay during cell cycle progression. Furthermore, it is possible that the administered dose of CHR-6494 (50 mg/kg) was not sufficient to inhibit HASPIN function, especially considering that the IC_50_ values of CHR-6494 are higher in breast cancer cell lines than in other cancer cell lines, such as HCT-116 and HeLa cells [22]. Although the administration conditions used have been shown to be effective in inhibiting tumor growth of colorectal cancer cells *in vivo* [22, 28], we thought that it might be worth investigating whether cancer progression is suppressed by other dose regimens of CHR-6494.

Taken together, our findings imply that although HASPIN is required for breast cancer cell proliferation, its partial inhibition by chemical agents might not be sufficient to suppress tumor growth *in vivo*. Development of more potent and selective HASPIN inhibitors and new biotechnological applications such as CRISPR interference that allow suppression of genetic transcription would be valuable in the treatment of breast cancer patients.

## Materials and methods

### Cell culture

MCF10A, SKBR3, MCF7, and MDA-MD-231 cells were obtained from the American Type Culture Collection (ATCC). MCF7 and MDA-MB-231 cells were maintained in Dulbecco’s modified Eagle’s medium. SK-BR-3 cells were cultured in RPMI-1640 medium. Cell growth media were supplemented with stable L-glutamine, 10% fetal bovine serum, and 1% penicillin-streptomycin. MCF10A cells were grown in DMEM/F12 medium containing 5% horse serum, 0.5 μg/mL hydrocortisone, 100 ng/mL cholera toxin, 10 μg/mL insulin, and 10 ng/mL recombinant epidermal growth factor (EGF), as described previously [37]. For doubling time measurement, we seeded approximately 10^5^ cells in 35-mm or 60-mm dishes and replaced the growth medium every 3 days. The numbers of cells of each cell line were counted in triplicate after staining with 0.5% trypan blue solution. After plotting the growth curve, we calculated the doubling time from the logarithmic phase of the growth curve.

### qRT-PCR

Total RNAs were purified using ISOGEN II (Nippon Gene), and reverse transcription was performed using a PrimeScript RT reagent kit (TaKaRa) according to the manufacturer’s instructions. qRT-PCR was performed using Roter-Gene Q (Qiagen) with THUNDERBIRD SYBR qPCR Mix (Toyobo). The mRNA levels of *HASPIN* were measured using human *HASPIN*-specific primers (forward: 5’-ACA AGT GGT GCT CCG TCC TCT T-3’; reverse: 5’-AGG ACC CTT CTG ACT GCA TTC C-3’) and normalized to the levels of *GAPDH* (forward: 5’-CAT GAG AAG TAT GAC AAC AGC CT-3’; reverse: 5’-AGT CCT TCC ACG ATA CCA AAG T-3’).

### Cell viability assay

CHR-6494 (372040-10MG, Lot: 2892867; Millipore) was dissolved at 10 mM in dimethyl sulfoxide (DMSO) and stored at -20°C. Cell viability was assessed using the XTT assay (#11465015001, Sigma) according to the manufacturer’s instructions. Cells were seeded in 96-well plates at a density of 2 × 10^4^ cells per well and allowed to attach for 24 h. Subsequently, cells were treated with different CHR-6494 concentrations (0.01–100 μM; eight replicates for each concentration). The XTT reagent was added 48 h after drug administration, and the absorbance was measured 1 h after adding the XTT reagent. IC_50_ values were determined using GraphPad Prism software (GraphPad).

### Immunoblotting

Cells were seeded in 60-mm dishes at a density of 5 × 10^5^ cells per dish. After 12 h of treatment with CHR-6494 or DMSO, cells were washed with phosphate-buffered saline (PBS) and then lysed in sodium dodecyl sulfate (SDS) sample buffer (50 mM Tris, pH6.8, 100 mM DTT, 2% SDS, 8% glycerol, 1 mM bromophenol blue). Proteins were separated by SDS-PAGE and transferred to PVDF membranes (Bio-Rad). After blocking with 5% skim milk in TBST, membranes were treated with anti-Histone H3 (phospho T3) (#ab78351; Abcam) or anti-GAPDH (#AM4300; Thermo Fisher Scientific) antibody. Chemiluminescence images were acquired with the LAS 4000 mini (GE Healthcare).

### Apoptosis and cell cycle analysis

Cells were seeded in 150-mm cell-culture dishes (3 × 10^6^ for MCF7 and SK-BR-3 cells; 2 × 10^6^ for MDA-MB-231 cells) and allowed to attach for 24 h. Subsequently, cells were treated with different CHR-6494 concentrations (0.5 or 1.0 M) or DMSO as a control. After 48 h, floating and attached cells were collected and washed with PBS. Aliquots of approximately 1 × 10^5^ cells were resuspended in 100 L of Annexin V binding buffer (BioLegend) containing 5 L FITC-Annexin V (BioLegend) and 10 L PI solution (Molecular Probes). After a 15-min incubation at room temperature in the dark, cells were resuspended in 400 L of Annexin V binding buffer. Stained cells were analyzed by flow cytometry (FACSCalibur; Becton Dickinson).

For cell cycle analysis, we fixed approximately 2 × 10^6^ cells in ice-cold 70% ethanol on ice for 30 min. Cells were washed twice with PBS and stained with 25 g/mL PI in a solution containing 50 g/mL RNase A (Sigma). To label the mitotic cells, cells were stained with 10 g/mL of Cy5-conjugated mouse anti-phospho-Ser/Thr-Pro MPM-2 antibody (# 16–220; Millipore Sigma) and PI. Stained cells were analyzed by flow cytometry (FACSCaliber [Fig 3A] or FACSCanto II [Fig 3B]; Becton Dickinson). The proportions of cells in each cell cycle stage were determined based on the PI fluorescence intensity profile of the cells using the Dean-Jett-Fox cell cycle modeling algorithm. The data were analyzed using FlowJo software.

### Xenograft mouse model

Seven-week-old BALB/c nu/nu female mice (Shimizu Laboratory Supplies) were used to establish tumor xenograft models. Mice were housed under pathogen-free conditions and acclimated for at least 1 week before the experiment. The experimental design was approved by the Animal Care and Use Committee of Kansai Medical University.

MDA-MB-231 cells were harvested at the exponential growth phase and resuspended in a 1:1 PBS–Matrigel (Corning) mixture at a density of 1 × 10^8^ cells/mL. Subsequently, cells were injected into the mammary fat pad of anesthetized nude mice (5 × 10^6^ cells per mouse). Body weight and tumor size were measured twice per week. Tumor volume (mm^3^) was calculated using the formula *V* = *D* × *d*^*2*^*/2*, where *D* is the long axis and *d* the short axis of the tumor. When tumors reached an average volume of 62 mm^3^ (2 weeks after injection), 14 mice harboring similar tumor sizes were randomly divided into two groups: control group (n = 7) treated with vehicle (10% DMEM/20% 2-hydroxypropyl-b-cyclodextrin [Sigma]) and treatment group (n = 7). Mice in the treatment group were intraperitoneally injected with 50 mg/kg of CHR-6494 diluted in 10% DMEM/20% 2-hydroxypropyl-cyclodextrin. Treatments were given in four cycles of 5 consecutive days over 35 days. At the end of the treatment, mice were sacrificed, and tumors were excised and weighted. The tumor mass was expressed as mean ± standard deviation (SD); significance was assessed using the Mann–Whiney U-test (GraphPad Prism software). In all analyses, *P* < 0.05 was taken to indicate statistical significance.

### Immunohistochemistry

Tumors were fixed overnight in PBS containing 4% paraformaldehyde and then incubated in PBS containing 20% sucrose for 24 h. Tumors were embedded in OCT (Sakura Finetek) and frozen in liquid nitrogen. Frozen tissues were used to prepare 14-m-thick sections with a cryostat, and sections were left to dry on glass slides. Subsequently, sections were washed with PBS three times and permeabilized with PBS containing 0.5% Triton-X100. After blocking, sections were stained with antibodies against CD31 (clone MEC13.3, 550274; BD Pharmingen) or LYVE-1 (clone E9VA4, sc-65647; Santa Cruz). After staining with the respective Alexa 555-labeled secondary antibodies (Thermo Fisher), cell nuclei were counterstained with Hoechst 33342 (Thermo Fisher). Stained sections were imaged using a BIOREVO BZ-9000 fluorescence microscope (KEYENCE). Tumor vessel sizes were measured using a BZ II analyzer (KEYENCE).

## Supporting information

S1 Fig (TIF)

## References

[1] Tanaka H, Yoshimura Y, Nozaki M, Yomogida K, Tsuchida J, Tosaka Y, et al. Identification and characterization of a haploid germ cell-specific nuclear protein kinase (Haspin) in spermatid nuclei and its effects on somatic cells. J Biol Chem. 1999;274(24):17049–57. 10.1074/jbc.274.24.17049 10358056

[2] Tanaka H, Iguchi N, Nakamura Y, Kohroki J, de Carvalho CE, Nishimune Y. Cloning and characterization of human haspin gene encoding haploid germ cell-specific nuclear protein kinase. Mol Hum Reprod. 2001;7(3):211–8. 10.1093/molehr/7.3.211 11228240

[3] Higgins JM. Structure, function and evolution of haspin and haspin-related proteins, a distinctive group of eukaryotic protein kinases. Cell Mol Life Sci. 2003;60(3):446–62. 10.1007/s000180300038 12737306 PMC11138542

[4] Dai J, Sultan S, Taylor SS, Higgins JM. The kinase haspin is required for mitotic histone H3 Thr 3 phosphorylation and normal metaphase chromosome alignment. Genes Dev. 2005;19(4):472–88. 10.1101/gad.1267105 15681610 PMC548948

[5] Kelly AE, Ghenoiu C, Xue JZ, Zierhut C, Kimura H, Funabiki H. Survivin reads phosphorylated histone H3 threonine 3 to activate the mitotic kinase Aurora B. Science. 2010;330(6001):235–9. 10.1126/science.1189505 20705815 PMC3177562

[6] Wang F, Dai J, Daum JR, Niedzialkowska E, Banerjee B, Stukenberg PT, et al. Histone H3 Thr-3 phosphorylation by Haspin positions Aurora B at centromeres in mitosis. Science. 2010;330(6001):231–5. 10.1126/science.1189435 20705812 PMC2967368

[7] Yamagishi Y, Honda T, Tanno Y, Watanabe Y. Two histone marks establish the inner centromere and chromosome bi-orientation. Science. 2010;330(6001):239–43. 10.1126/science.1194498 20929775

[8] Han X, Kuang T, Ren Y, Lu Z, Liao Q, Chen W. Haspin knockdown can inhibit progression and development of pancreatic cancer in vitro and vivo. Exp Cell Res. 2019;385(1):111605. 10.1016/j.yexcr.2019.111605 31493385

[9] Higgins JM. The Haspin gene: location in an intron of the integrin alphaE gene, associated transcription of an integrin alphaE-derived RNA and expression in diploid as well as haploid cells. Gene. 2001;267(1):55–69. 10.1016/s0378-1119(01)00387-0 11311556

[10] Melms JC, Vallabhaneni S, Mills CE, Yapp C, Chen JY, Morelli E, et al. Inhibition of Haspin Kinase Promotes Cell-Intrinsic and Extrinsic Antitumor Activity. Cancer Res. 2020;80(4):798–810. 10.1158/0008-5472.CAN-19-2330 31882401 PMC7029677

[11] Zhu D, Gu X, Lin Z, Yu D, Wang J, Li L. HASPIN is involved in the progression of gallbladder carcinoma. Exp Cell Res. 2020;390(2):111863. 10.1016/j.yexcr.2020.111863 31987787

[12] Ye Z, Zhang Z, Fang L, Tian D, Liu X. Bioinformatic analysis reveals GSG2 as a potential target for breast cancer therapy. Open Life Sciences. 2019;14(1):688–98. 10.1515/biol-2019-0078 33817208 PMC7874749

[13] Amoussou NG, Bigot A, Roussakis C, Robert JH. Haspin: a promising target for the design of inhibitors as potent anticancer drugs. Drug Discov Today. 2018;23(2):409–15. 10.1016/j.drudis.2017.10.005 29031622

[14] Patnaik D, Jun Xian, Glicksman MA, Cuny GD, Stein RL, Higgins JM. Identification of small molecule inhibitors of the mitotic kinase haspin by high-throughput screening using a homogeneous time-resolved fluorescence resonance energy transfer assay. J Biomol Screen. 2008;13(10):1025–34. 10.1177/1087057108326081 18978305 PMC2615828

[15] Cuny GD, Robin M, Ulyanova NP, Patnaik D, Pique V, Casano G, et al. Structure-activity relationship study of acridine analogs as haspin and DYRK2 kinase inhibitors. Bioorg Med Chem Lett. 2010;20(12):3491–4. 20836251 PMC3118465

[16] Cuny GD, Ulyanova NP, Patnaik D, Liu JF, Lin X, Auerbach K, et al. Structure-activity relationship study of beta-carboline derivatives as haspin kinase inhibitors. Bioorg Med Chem Lett. 2012;22(5):2015–9. 10.1016/j.bmcl.2012.01.028 22335895 PMC3288743

[17] De Antoni A, Maffini S, Knapp S, Musacchio A, Santaguida S. A small-molecule inhibitor of Haspin alters the kinetochore functions of Aurora B. J Cell Biol. 2012;199(2):269–84. 10.1083/jcb.201205119 23071153 PMC3471222

[18] Karanika E, Soupsana K, Christogianni A, Stellas D, Klinakis A, Politou AS, et al. Haspin-dependent and independent effects of the kinase inhibitor 5-Iodotubercidin on self-renewal and differentiation. Sci Rep. 2020;10(1):232. 10.1038/s41598-019-54350-4 31937797 PMC6959359

[19] Wang P, Hua X, Bryner YH, Liu S, Gitter CB, Dai J. Haspin inhibition delays cell cycle progression through interphase in cancer cells. J Cell Physiol. 2020;235(5):4508–19. 10.1002/jcp.29328 31625162 PMC7018545

[20] Balzano D, Santaguida S, Musacchio A, Villa F. A general framework for inhibitor resistance in protein kinases. Chem Biol. 2011;18(8):966–75. 10.1016/j.chembiol.2011.04.013 21867912

[21] Wang F, Ulyanova NP, Daum JR, Patnaik D, Kateneva AV, Gorbsky GJ, et al. Haspin inhibitors reveal centromeric functions of Aurora B in chromosome segregation. J Cell Biol. 2012;199(2):251–68. 10.1083/jcb.201205106 23071152 PMC3471242

[22] Huertas D, Soler M, Moreto J, Villanueva A, Martinez A, Vidal A, et al. Antitumor activity of a small-molecule inhibitor of the histone kinase Haspin. Oncogene. 2012;31(11):1408–18. 10.1038/onc.2011.335 21804608 PMC3312407

[23] Han L, Wang P, Sun Y, Liu S, Dai J. Anti-Melanoma Activities of Haspin Inhibitor CHR-6494 Deployed as a Single Agent or in a Synergistic Combination with MEK Inhibitor. J Cancer. 2017;8(15):2933–43. 10.7150/jca.20319 28928884 PMC5604444

[24] Hu Z, Fan C, Oh DS, Marron JS, He X, Qaqish BF, et al. The molecular portraits of breast tumors are conserved across microarray platforms. BMC Genomics. 2006;7:96. 10.1186/1471-2164-7-96 16643655 PMC1468408

[25] Voduc KD, Cheang MC, Tyldesley S, Gelmon K, Nielsen TO, Kennecke H. Breast cancer subtypes and the risk of local and regional relapse. J Clin Oncol. 2010;28(10):1684–91. 10.1200/JCO.2009.24.9284 20194857

[26] Chen Y, Fu D, Zhao H, Cheng W, Xu F. GSG2 (Haspin) promotes development and progression of bladder cancer through targeting KIF15 (Kinase-12). Aging (Albany NY). 2020;12(10):8858–79. 32439830 10.18632/aging.103005 PMC7288960

[27] Higgins JM. Haspin: a newly discovered regulator of mitotic chromosome behavior. Chromosoma. 2010;119(2):137–47. 10.1007/s00412-009-0250-4 19997740 PMC2839057

[28] Tanaka H, Wada M, Park J. HASPIN kinase inhibitor CHR-6494 suppresses intestinal polyp development, cachexia, and hypogonadism in Apcmin/+ mice. Eur J Cancer Prev. 2019. 10.1097/CEJ.0000000000000562 PMC7531494 31833958

[29] Dai J, Sullivan BA, Higgins JM. Regulation of mitotic chromosome cohesion by Haspin and Aurora B. Dev Cell. 2006;11(5):741–50. 10.1016/j.devcel.2006.09.018 17084365

[30] Shimada M, Goshima T, Matsuo H, Johmura Y, Haruta M, Murata K, et al. Essential role of autoactivation circuitry on Aurora B-mediated H2AX-pS121 in mitosis. Nat Commun. 2016;7:12059. 10.1038/ncomms12059 27389782 PMC4941122

[31] Kang TH, Park DY, Choi YH, Kim KJ, Yoon HS, Kim KT. Mitotic histone H3 phosphorylation by vaccinia-related kinase 1 in mammalian cells. Mol Cell Biol. 2007;27(24):8533–46. 10.1128/MCB.00018-07 17938195 PMC2169395

[32] Nezu J, Oku A, Jones MH, Shimane M. Identification of two novel human putative serine/threonine kinases, VRK1 and VRK2, with structural similarity to vaccinia virus B1R kinase. Genomics. 1997;45(2):327–31. 10.1006/geno.1997.4938 9344656

[33] Santos CR, Rodriguez-Pinilla M, Vega FM, Rodriguez-Peralto JL, Blanco S, Sevilla A, et al. VRK1 signaling pathway in the context of the proliferation phenotype in head and neck squamous cell carcinoma. Mol Cancer Res. 2006;4(3):177–85. 10.1158/1541-7786.MCR-05-0212 16547155

[34] Moura DS, Campillo-Marcos I, Vazquez-Cedeira M, Lazo PA. VRK1 and AURKB form a complex that cross inhibit their kinase activity and the phosphorylation of histone H3 in the progression of mitosis. Cell Mol Life Sci. 2018;75(14):2591–611. 10.1007/s00018-018-2746-7 29340707 PMC6003988

[35] Wiebe MS, Nichols RJ, Molitor TP, Lindgren JK, Traktman P. Mice deficient in the serine/threonine protein kinase VRK1 are infertile due to a progressive loss of spermatogonia. Biol Reprod. 2010;82(1):182–93. 10.1095/biolreprod.109.079095 19696012 PMC2802121

[36] Wang F, Ulyanova NP, van der Waal MS, Patnaik D, Lens SM, Higgins JM. A positive feedback loop involving Haspin and Aurora B promotes CPC accumulation at centromeres in mitosis. Curr Biol. 2011;21(12):1061–9. 10.1016/j.cub.2011.05.016 21658950 PMC3118923

[37] Fukuda S, Nishida-Fukuda H, Nanba D, Nakashiro K, Nakayama H, Kubota H, et al. Reversible interconversion and maintenance of mammary epithelial cell characteristics by the ligand-regulated EGFR system. Sci Rep. 2016;6:20209. 10.1038/srep20209 26831618 PMC4735799

