# Supplementary figures and images for: Evaluation of the antiproliferative effects of the HASPIN inhibitor CHR-6494 in breast cancer cell lines
Source: PLoS One. 2021 Apr 14;16(4):e0249912. doi: 10.1371/journal.pone.0249912 (PMC8046223; doi:10.1371/journal.pone.0249912)

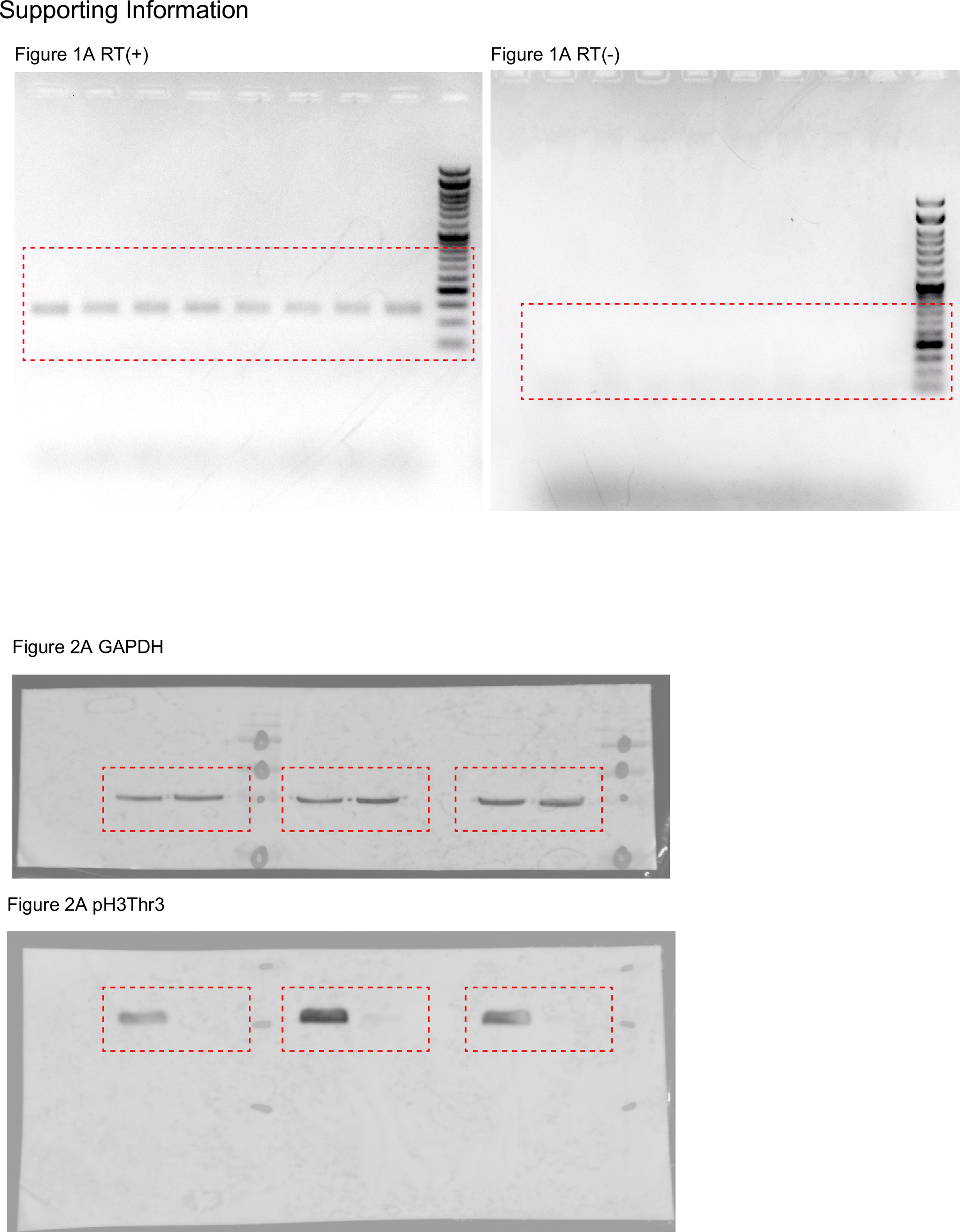

Supplement: S1 Fig — (TIF) [file pone.0249912.s001.tif]
